# Reliability and Validity of the Japanese Version of the Implementation Leadership Scale for Nurse Managers and Staff Nurses: A Cross-Sectional Study

**DOI:** 10.1155/2023/4080434

**Published:** 2023-10-23

**Authors:** Masatoshi Saiki, Ai Tomotaki, Hiroki Fukahori, Takeshi Yamamoto, Masakazu Nishigaki, Chiyo Matsuoka, Emi Yasuda, Ikuko Sakai

**Affiliations:** ^1^Department of Advanced Clinical Nursing, Frontier Clinical Nursing, Graduate School of Nursing, Chiba University, Chiba, Japan; ^2^Faculty of Nursing, School of Medicine, Tokai University, Kanagawa, Japan; ^3^Faculty of Nursing and Medical Care, Keio University, Tokyo, Japan; ^4^Department of Nursing, School of Health Sciences, Sapporo Medical University, Hokkaido, Japan; ^5^School of Nursing at Narita, International University of Health and Welfare, Tokyo, Japan; ^6^Department of Nursing, Faculty of Nursing and Rehabilitation, Konan Women's University, Hyogo, Japan; ^7^Department of Health and Welfare Services, National Institute of Public Health, Saitama, Japan

## Abstract

**Background:**

Strategic leadership is key to implementing evidence-based practice (EBP). Evaluating the readiness and processes necessary to implement EBP using the Japanese version of the implementation leadership scale (ILS) may be useful to systematically promote the implementation of EBP in Japan. This study aimed to evaluate the reliability and validity of the Japanese version of the ILS for nurse managers and staff nurses.

**Methods:**

Data were collected in a cross-sectional study. The original ILS was translated into Japanese and back-translated into English. Clinical nurses reviewed it and confirmed its face validity. We distributed a web questionnaire to 119 nurse managers and 2,858 staff nurses working at three university hospitals in Japan's metropolitan areas. Construct validity was assessed for nursing managers and staff nurses, respectively, using confirmatory factor analysis. Known-group validity for nurse managers was assessed by verifying differences in ILS scores by educational background and experience of learning EBP and working on EBP. We evaluated reliability using Cronbach's alpha and test-retest reliability.

**Results:**

The response rates for nurse managers and staff nurses were 56.3% and 16.9%, respectively. Data from 67 nursing managers and 484 staff nurses were analyzed, excluding duplicate responses. Confirmatory factor analyses of both samples supported the four-factor structure of ILS. The ILS total score of nurse managers with experience learning EBP or experience working on EBP was statistically significantly higher than that of those with no experience, and known-group validity was supported. Across both samples, internal consistency reliability was strong (Cronbach's alpha: 0.91–0.97) and test-retest reliability was moderate.

**Conclusion:**

This study illustrated the reliability and validity of Japanese versions of the ILS for both nurse managers and staff nurses. This study enabled international comparisons of the leadership required for EBP implementation and may support the development of intervention programs and strategies to promote EBP's implementation in different countries. *Trial Registration*. UMIN Clinical Trials Registry (UMIN-CTR). This trail is registered with UMIN000045782.

## 1. Introduction

Evidence-based practice (EBP) is the integration of the best available research evidence with clinical expertise and individual patient values and circumstances [1]. Successful implementation of EBP is expected to result in more effective healthcare service delivery, reduced costs, and increased clinician and patient satisfaction [2–4]. Hence, EBP implementation is widely accepted worldwide as a priority to promote quality clinical practice and optimal patient outcomes [5, 6].

Previous studies have indicated that EBP is not consistently used by nurses in clinical settings [7, 8]. EBP implementation at the unit level has various obstacles, such as fewer opportunities to learn about EBP implementation [9] and nursing managers' passive attitude toward EBP implementation [10]. EBP is challenging for staff nurses to implement on their own. Nurse managers play an important role in all processes of EBP implementation because they can influence the organizational climate of the workplace, the degree of cooperation among team members, and the attitudes of staff nurses toward EBP [11–14]. Therefore, EBP implementation in the unit requires not only the effort of individual staff nurses but also a proactive approach by nurse managers. Nurse manager leadership is an important factor in successful EBP implementation [15, 16].

Evidence suggests a relationship between EBP implementation and managerial leadership. Supervisors' transformational leadership and transactional leadership are associated with followers' positive attitudes toward the adoption of EBP [17, 18]. Nurse manager leadership in EBP implementation is defined as a multidimensional process that influences staff, their environment, and the organizational infrastructure to facilitate the integration of evidence into clinical practice [19]. Nurse managers are required to strategically create an EBP implementation environment and take action to promote the implementation and sustainability of EBP. Strategic leadership involves anticipating organizational change, making strategic decisions, and managing the change process [19, 20]. A meta-analysis conducted by Hong et al. [13] confirmed that strategic leadership is advantageous for specific organizational change initiatives. Hence, the strategic leadership of nurse managers can be useful for EBP implementation.

Based on theory and previous research on EBP implementation, leadership, and organizational membership, Aarons et al. [11] developed the implementation leadership scale (ILS), a psychometric scale that assesses the strategic leadership demonstrated by leaders in EBP implementation. The scale consists of 12 items and four subscales, and its brevity makes it appropriate for busy healthcare settings. The ILS has both leader and staff versions, which is useful in that it allows evaluation from multiple perspectives. The ILS, which was originally in English, has been translated into Chinese, Greek, and Norwegian, and its reliability and validity have been confirmed [3, 21, 22]. Previous studies reported that the ILS was associated with the multifactor leadership questionnaire (MLQ) [11, 21] and the implementation climate scale [23] as measures of convergent validity and less associated with the evidence-based practice attitude scale (EBPAS) [21, 23] and organizational climate measure [3, 11] as measures of discriminant validity. Moreover, the ILS has been used to develop intervention programs [24] and as an evaluative tool for educational intervention research [25]. As mentioned above, the ILS has been shown to be associated with multiple psychological measures and has been used in intervention research, but its psychometric properties (i.e., which individual characteristics are associated with the ILS scores) have not been adequately tested [3, 26].

The Japanese version of the ILS will allow the exploration of relevant factors, the development and evaluation of educational intervention programs, and international comparisons. As in many other countries, the implementation of EBP in Japan is of growing interest to ensure the best possible care delivery by integrating research evidence and clinical practice. However, EBP implementation and research in Japan are ongoing and developing slowly [27]. The Japanese version of the ILS is expected to promote research on EBP implementation in Japan's healthcare settings. This study aimed to evaluate the translated ILS factor structure and psychometric properties of nurse managers and staff nurses in a Japanese nursing context.

## 2. Materials and Methods

### 2.1. Study Design

This was a cross-sectional study for the psychometric testing of the Japanese version of the ILS. The original ILS has two versions: one for leaders to measure their leadership, and one for staff to measure the leadership of the head of their unit.

### 2.2. The Japanese Version of ILS

The original ILS was translated into Japanese by a professional English translator who was not a nursing expert, and the translation was reviewed and confirmed by the authors. Face validity was also assessed by 20 clinical nurses. Subsequently, the ILS was back-translated into English by the authors, and permission was obtained from the original authors.

The validity of the ILS in the Japanese population was assessed in terms of content validity, construct validity, and criterion-related validity (known-group validity). Reliability was assessed using internal consistency and the test-retest method.

### 2.3. Participants and Sample Size

The participants were nurses working at three university hospitals in the Greater Tokyo area of Japan. Using convenience sampling, we selected hospitals where ILS scores were expected to vary among subjects. The inclusion criterion for participants was nurse managers and staff nurses working at the target facility, and the exclusion criterion was the head of the nursing department.

In setting the sample size, several issues were considered. First, the ILS is available in two types: one for staff and one for leaders; therefore, a minimum of 100 cases were required for each analysis [28]. Second, the response rate in previous studies on Japanese nurse managers was approximately 50% [29, 30]. Third, the selection of units and staff may raise issues of subject selection bias and ethical considerations. Finally, the results from only one facility may have biased responses. We estimated approximately 50 nursing managers per hospital and a 50 percent response rate. We planned to recruit participants from four hospitals to reach the needed sample size of nurse managers, but we refrained from recruiting one of them due to the COVID-19 pandemic.

For the survey of staff nurses, we were concerned about the arbitrary selection of staff and the possibility of discouraging voluntary participation. Therefore, we targeted all staff nurses in approximately 150 departments at the three facilities to which the targeted nursing managers belonged and estimated a maximum of 3,000 survey forms.

### 2.4. Data Collection

The study was conducted from October 2021 to February 2022. We requested for their cooperation in the study and obtained consent from the head of the nursing department at each facility. An online survey tool was used, and leaflets with QR codes were distributed to 119 nurse managers and 2,858 staff nurses in the three facilities. The remainder of the study was sent to nurse managers in January 2022. For test-retest reliability, participants were asked to answer the survey again at least two weeks after the completion of the initial survey.

### 2.5. Instruments

#### 2.5.1. Demographics

Participants were asked about their gender, age, academic background, years of experience as clinical nurses, and years of experience working in the unit.

#### 2.5.2. The Implementation Leadership Scale (ILS)

This scale measures unit-level leaders' leadership in implementing EBPs and consists of 12 items and four subscales (proactive leadership, knowledgeable leadership, supportive leadership, and perseverant leadership). This scale has two versions, one for leaders and one for staff. The leader version is a self-assessment, whereas the staff version is an evaluation of the leader's leadership by others. In the original ILS, because the target of each version is evaluated differently, the items are written with “I” in the leader's version and the name of the leader to be evaluated in the staff version. Respondents rated the items on a 5-point Likert scale ranging from 0 (not at all) to 4 (very great extent). The scale scores were calculated as the mean of each subscale and all items.

#### 2.5.3. Experiences in Learning EBP and Working on EBP Implementation

The participants were asked about their experiences in learning EBP and working on EBP implementation, respectively, with “No,” “Yes,” and “Unsure” as possible answers.

### 2.6. Statistical Analysis

We tested the construct validity, known-group validity, internal consistency, and test-retest reliability of the ILS using the following statistical methods: Construct validity was tested using confirmatory factor analysis (CFA) and the following model fit indexes: comparative fit index (CFI), Tucker-Lewis index (TLI), root mean square error of approximation (RMSEA), and standardized root mean square residual (SRMR). CFI and TLI values greater than 0.90, RMSEA values less than 0.10, and SRMR values less than 0.09 indicate an acceptable fit of the model [31, 32]. This study assumed four-factor structure as the original study [11]. Furthermore, since staff nurses working in the same unit have the same leader, the CFA was conducted considering a multilevel nested data structure for staff nurses.

We tested for known-group validity using the nurse managers' ILS scores. Nurse leaders' graduate-level education, years of leadership experience, and completion of leadership courses are important factors that positively influence their leadership for EBP implementation [33]. Previous research has suggested that nurses with a master's degree or higher had higher knowledge and skills of EBP and more positive attitudes about EBP implementation than nurses with diploma and baccalaureate degrees [9, 34, 35]. Based on these findings, we hypothesized that nurse managers with postgraduate degrees would have higher ILS scores than those with other educational levels. The nurse managers' educational backgrounds were divided into three groups: (a) high school/vocational school/junior college, (b) university, and (c) graduate school. The Kruskal–Wallis test with a post hoc Bonferroni–Dunn test was used to examine our hypothesis. Furthermore, nurse managers who learned EBP reported enhanced leadership in EBP implementation [36]. Mentoring by nurses with adequate EBP experience facilitates EBP implementation [10, 37]. These results imply adequate EBP learning and working by unit leaders, which probably strengthens their leadership for the implementation of EBP. Therefore, we hypothesized that nurse managers with experiences in learning and working on EBP would have higher ILS scores than those without such experiences. The Mann–Whitney *U* test was used to verify the prediction that nurses with experiences in learning and working on EBP implementation would score higher than those without such experiences.

Internal consistency was assessed using Cronbach's alpha for each subscale. The intraclass correlation coefficient (ICC): ICC (1) and average within-group correlation (a_wg_) were calculated as indexes of agreement to determine whether staff nurses' scores were a unit-level component. The ICC (1) was interpreted based on 0.12 proposed by James [38], although there is no clear criterion, with higher values indicating higher group-level agreeableness. Values of a_wg_ greater than 0.60 represent acceptable agreement [39].

In the test-retest method, ICC_(2,1)_ was calculated for each subscale of the ILS. Values of ICC 0.5– 0.75 was moderate while ICC< 0.5 was poor [40]. Weighted kappa coefficients were calculated for agreement for each item. The agreement levels were suggested as follows: 0–0.2 (poor), 0.2–0.4 (fair), 0.4–0.6 (moderate), 0.6–0.8 (substantial), and 0.8–1.0 (almost perfect) [41, 42]. The analyses were conducted using IBM SPSS ver. 28.0 and Mplus ver. 8.0. ICC (1), and a_wg_ were calculated using the “multilevel” package [43] in the statistical software R ver 4.2.0. The significance level was set at 5%.

### 2.7. Ethical Consideration

This study was approved by the Institutional Ethics Review Board of the Chiba University Graduate School of Nursing. Participants accessed the explanatory document via the web page individually from the study participation leaflet. After reading it, consent was obtained at the beginning of the study. Participants were given 500 JPY in electronic money as an incentive after responding to the first survey.

## 3. Results

### 3.1. Content Validity

In the Japanese version of the ILS for staff, for the sake of generality, the name of the leader of an item was set as the “leader of the department.” The content of all items in the Japanese version of the ILS was confirmed through discussions among researchers, and consensus was reached, which was judged to ensure content validity.

### 3.2. Questionnaire Participants and Demographic Data

**Figure 1:**
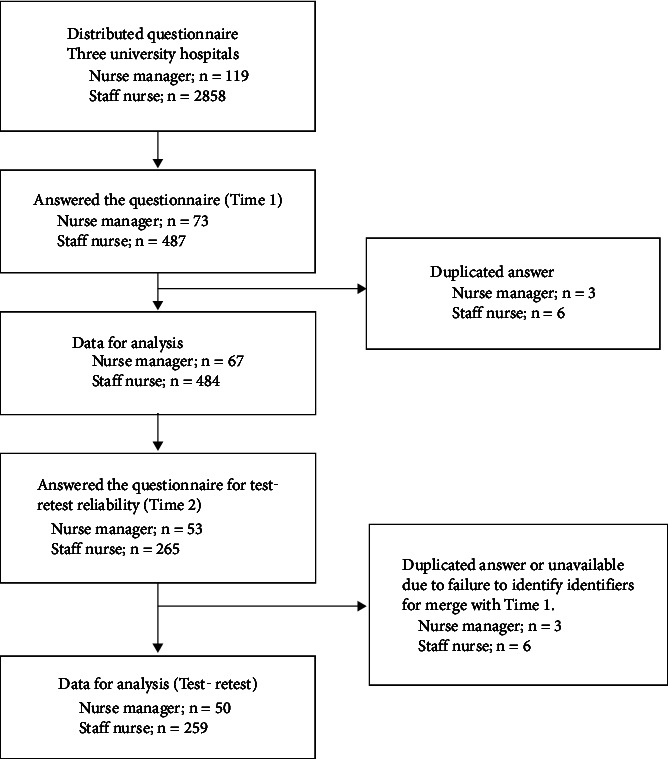
Participants flow.

The flow diagram of the study participants is given in Figure 1. In total, 67 nurse manager and 484 staff data were analyzed (response rate; nurse manager: 56.3%, staff: 16.9%), and the response rate for each hospital ranged from 16.8 to 21.5%. Of these, 53 nurse managers and 265 staff nurses responded to the retest survey. The participant demographics are shown in Table 1. More than 90% of the participants were female. 97% of nurse managers were over 40 years old, and 77.5% of staff nurses were under 40 years old.

### 3.3. Internal Consistency and Group-Agreement Indexes

Table 2 shows the ILS items and means, SD, reliabilities, and aggregation statistics. Cronbach's *α* ranged from 0.91 to 0.97 for nurse managers and from 0.94 to 0.97 for staff nurses. For staff nurses, the within-group agreement index a_wg_ ranged from 0.47 to 0.56, all items below the criteria of 0.60. ICCs ranged from 0.16 to 0.22, which are generally acceptable values.

### 3.4. Construct Validity

**Figure 2:**
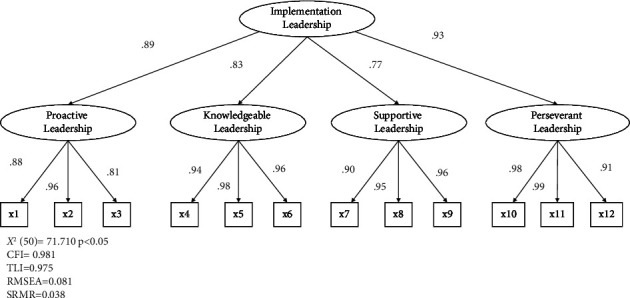
Second-order confirmatory factor analysis for the ILS of the nurse managers.

**Figure 3:**
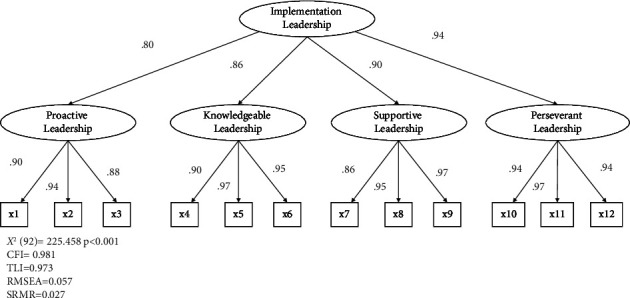
Second-order confirmatory factor analysis for the ILS of the staff nurses.

CFA validated the original model, consisting of 12 items and four factors. The nurse manager data showed CFI = 0.981, TLI = 0.975, RMSEA = 0.081, and SRMR = 0.038, indicating an acceptable model fit (Figure 2). First-order factor loadings ranged from 0.81 to 0.99, and second-order factor loadings ranged from 0.77 to 0.93. The staff nurse data showed CFI = 0.981, TLI = 0.973, RMSEA = 0.057, and SRMR = 0.027, indicating an acceptable model fit (Figure 3). First-order factor loadings ranged from 0.86 to 0.97, second-order factor loadings ranged from 0.80 to 0.94. The Japanese version of the ILS supports the original factor structure for both leaders and staff.

### 3.5. Known-Group Validity

The ILS total scores of nurse managers were compared in three groups by education level: (a) high school/vocational school/junior college, (b) university, and (*c*) graduate school. Mean (SD: standard deviation) scores for a, b, and c were 1.25 (0.72), 1.54 (0.80), and 2.05 (0.78), respectively (*H* = 9.557, *p* = 0.008), with *a* < *c* (*p* = 0.006) for multiple comparisons.

The ILS total scores of nurse managers were also compared based on their experience learning EBP and working on EBP implementation. Those with experience learning EBP scored 1.89 (0.84) and those with no experience scored 1.03 (0.57) (*U* = −3.452, *p* < 0.001). Those with experience working on EBP scored 2.04 (0.85) and those with no experience scored 1.00 (0.48) (*U* = −4.229, *p* < 0.001).

### 3.6. Test-Retest Reliability

The ICC_(2,1)_ values of the nurse managers for the subscales were as follows: proactive leadership, 0.58; knowledgeable leadership, 0.79; supportive leadership, 0.71; perseverant leadership, 0.67; and total scale, 0.80. For the staff nurses, the ICC_(2,1)_ values for the subscales were as follows: proactive leadership, 0.64; knowledgeable leadership, 0.72; supportive leadership, 0.72; perseverant leadership, 0.74; and total scale, 0.78. All the ICC values were moderate according to the criterion.

The nurse managers' weighted kappa coefficient values for each item in the subscales were within the following ranges: proactive leadership, 0.22–0.47; knowledgeable leadership, 0.56–0.60; supportive leadership, 0.47–0.50; and perseverant leadership, 0.38–0.56. For the staff nurses, the weighted kappa coefficient values ranged as follows: proactive leadership, 0.41–0.43; knowledgeable leadership, 0.50–0.55; supportive leadership, 0.48–0.57; and perseverant leadership, 0.52–0.57 (Table 3). The weighted Kappa coefficient values were fair in two items and moderate in the rest of the items, according to the criterion.

## 4. Discussion

### 4.1. Validity of the Japanese Version of the ILS

This study translated the ILS into Japanese and confirmed the validity and reliability of the nurse manager and staff nurse versions. CFA, assuming a four-factor model [11], showed a moderate to good fit with the Japanese version of the ILS, suggesting its construct validity. Previous validation studies of translated ILS have supported the original four-factor model [3, 21, 22], and this factor structure was consistent across studies in different contexts.

Testing the known-group validity of the nurse managers' version of the scale showed a statistically significant group difference between (a) high school/vocational school/junior college and (c) graduate school. Nurse managers whose last education was (b) college showed no statistically significant group differences compared with the other groups, but the mean of the scale was confirmed to increase according to educational background. Previous studies have reported that factors associated with an individual's EBP implementation included EBP training, university position, higher education, professionalism, and belief in EBP [8], and our results mostly support this. These results confirm the known-group validity of the scale for nurse managers. However, this study did not examine the staff's known-group validity. The relationship with the nurse manager and experience of working together may be relevant, and further validation is needed in the future.

This study reveals differences in ILS scores according to nurse managers' attributes. This knowledge can assist in evaluating the implementation of leadership and developing intervention programs for EBP implementation.

### 4.2. Reliability of the Japanese Version of the ILS

Cronbach's *α*, indicating internal consistency, was greater than 0.9 for each subscale for both nurse managers and staff nurses. The Kappa coefficient, which indicates temporal stability, shows an overall moderate agreement was confirmed. In addition, the ICC of the ILS showed moderate values, confirming temporal stability. With these results, the validity and reliability of the Japanese version of the ILS were confirmed for both nurse managers and staff nurses. Whereas a few items in the nurse manager version showed particularly low temporal stability. These items might have influenced responses at each point in terms of the interpretation of adjectives such as “clear department standard” and “critical issues” in the wording. Therefore, several items need to be examined more closely to improve validity and reliability.

### 4.3. Linking Evidence to Action

The Japanese version of ILS allows us to understand the relationship between nurse managers' strategic leadership and EBP implementation in units, as well as to make international comparisons of leadership for EBP implementation. Moreover, ILS was found to be compatible with the Ottawa Model of Implementation Leadership, a theoretical model of implementation leadership [24], thus providing a foundation for intervention research. In future studies, the ILS can be used as an evaluation instrument for educational interventions targeting nurse managers in EBP implementation and compound interventions in facilities.

### 4.4. Limitation

The participants were nurses from three university hospitals in a metropolitan area of Japan, which may have biased the population. In addition, university hospitals may have more nurses with experience learning or working with EBP than other hospitals because of their role as educational and research institutions. Therefore, this sample may not necessarily be representative of all clinical nurses in Japan, and additional validation using other samples is needed. Furthermore, this study did not examine associations between the ILS and other scales with similar concepts; further validation of the relationships between other indicators related to leadership is needed in the Japanese context.

## 5. Conclusion

This study developed Japanese versions of the ILS for nurse managers and staff nurses. Our findings suggest that it is a valid and reliable measurement of leadership in EBP implementation. The findings of this study may contribute to increasing the reliability of assessing EBP implementation in Japan since the ILS is considered an effective tool for measuring leadership when implementing EBP.

## Figures and Tables

**Table 1:** Demographic data.

	Nurse manager, *n* = 67		Staff, *n* = 484	
	*n*	(%)	*n*	(%)
Gender				
Female	65	97.0	445	91.9
Male	1	1.5	26	5.4
Unknown	1	1.5	13	2.7
Age group				
Below 25	0	0.0	121	25.0
26–30	0	0.0	121	25.0
31–40	2	3.0	133	27.5
41–50	28	41.8	79	16.3
Above 51	37	55.2	30	6.2
Academic background				
Bachelor's degree	25	37.3	352	72.7
Master's degree	15	22.4	39	8.1
Doctoral degree	0	0.0	3	0.6
No degree^※^	27	40.3	90	18.6
Years of experience as clinical nurses				
Below 1	0	0.0	56	11.6
2 or 3	0	0.0	82	16.9
4–10	0	0.0	161	33.3
Above 11	67	100.0	185	38.2
Years of experience working in the unit				
Below 1	5	7.5	76	15.7
2 or 3	16	23.9	118	24.4
4–10	23	34.3	178	36.8
Above 11	23	34.3	112	23.1
Learning experience of EBP				
No	18	26.9	176	36.4
Yes	35	52.2	118	24.4
Unsure	14	20.9	190	39.3
Experience of working on EBP				
No	24	35.8	182	37.6
Yes	28	41.8	110	22.7
Unsure	15	22.4	192	39.7

Notes: ^※^It includes high school, vocational school, and junior college.

**Table 2:** Implementation leadership scale, subscale, and item statistics.

	Nurse manager, *n* = 67			Staff, *n* = 484				
	Mean	SD	*α*	Mean	SD	ICC(1)	a_wg_	*α*
1. Proactive leadership	1.33	1.04	0.91	1.57	1.06	0.21	0.54	0.94
(1) Developed a plan to facilitate EBP implementation	1.15	0.96		1.48	1.06		0.51	
(2) Removed obstacles to implementation of EBP	0.91	0.95		1.29	1.08		0.52	
(3) Established clear standards for implementation of EBP	1.13	0.90		1.45	1.01		0.54	
2. Knowledgeable leadership	1.54	0.91	0.97	1.93	1.12	0.19	0.50	0.97
(4) Is knowledgeable about EBP	1.49	0.94		1.85	1.13		0.53	
(5) Is able to answer staff questions about EBP	1.42	0.94		1.86	1.12		0.47	
(6) Knows what he/she is talking about when it comes to EBP	1.48	0.91		1.88	1.09		0.47	
3. Supportive leadership	2.15	0.94	0.96	2.00	1.15	0.22	0.56	0.96
(7) Recognizes and appreciates employee efforts	1.93	0.96		1.87	1.17		0.49	
(8) Supports employee efforts to learn more about EBP	1.99	0.96		1.88	1.16		0.56	
(9) Supports employee efforts to use EBP	2.02	0.91		1.92	1.11		0.56	
4. Perseverant leadership	1.58	0.94	0.97	1.79	1.13	0.16	0.52	0.97
(10) Perseveres through the ups and downs of implementing	1.54	0.96		1.78	1.11		0.51	
(11) Carries on through the challenges of implementing EBP	1.43	1.00		1.82	1.12		0.51	
(12) Reacts to critical issues regarding implementation of EBP	1.52	0.94		1.80	1.08		0.51	
Implementation leadership scale total	1.54	0.81	0.96	1.76	0.98	0.22		0.97

*Note.* Range [0–4]; SD: standard deviation.

**Table 3:** Test-retest reliability.

	Nurse manager, *n* = 50		Staff, *n* = 259	
	Weighted kappa coefficient	ICC_(2,1)_	Weighted kappa coefficient	ICC_(2,1)_
Proactive leadership		0.58		0.64
(1) Developed a plan to facilitate EBP implementation	0.43		0.41	
(2) Removed obstacles to implementation of EBP	0.47		0.43	
(3) Established clear standards for implementation of EBP	0.22		0.44	
Knowledgeable leadership		0.79		0.72
(4) Is knowledgeable about EBP	0.56		0.55	
(5) Is able to answer staff questions about EBP	0.60		0.54	
(6) Knows what he/she is talking about when it comes to EBP	0.59		0.50	
Supportive leadership		0.71		0.72
(7) Recognizes and appreciates employee efforts	0.47		0.48	
(8) Supports employee efforts to learn more about EBP	0.50		0.57	
(9) Supports employee efforts to use EBP	0.50		0.56	
Perseverant leadership		0.67		0.74
(10) Perseveres through the ups and downs of implementing	0.47		0.52	
(11) Carries on through the challenges of implementing EBP	0.44		0.57	
(12) Reacts to critical issues regarding implementation of EBP	0.38		0.56	
Implementation leadership scale total		0.80		0.78

## Data Availability

The datasets generated and/or analyzed during the current study are not publicly available due to the risk of identifying participants, but extracts are available from the corresponding author on reasonable request.

## Supplementary Materials

Supplementary Materials Supplementary Table S1: Framework for strengthening the reporting of observational studies in epidemiology (STROBE) statement.
